# Improving treatment outcomes with an eating disorder symptom-specific feedback tool

**DOI:** 10.1186/2050-2974-3-S1-O41

**Published:** 2015-11-23

**Authors:** Prudence Hepple, Sue Byrne, Karina Allen

**Affiliations:** 1 University of Western Australia, Australia; 2 Centre for Clinical Interventions, Australia

## 

Statistical methods of clinical decision making are necessary in our practice - clinical judgment alone is error-prone and often inaccurate. Using a database of female eating disorder (ED) outcome data (n=550) from the Centre for Clinical Interventions, trajectories of ED symptom change (using a validated 8-item version of the EDE-Q) were developed with linear mixed model analyses in MPlus for clients who did and did not achieve remission over the course of treatment. These symptom trajectories are able to be utilised as a clinical decision making tool to compare individual patients' weekly progress with expected progress, to assist clinicians in detecting patients who are at risk for a poor treatment outcome. From this, there is also scope to create a symptom-based feedback tool with reliable change indices to determine whether a client has achieved clinically significant improvement, experienced no change, or has deteriorated. The feedback tool developed in this study will be implemented into a randomised controlled trial to examine whether ED symptom feedback can improve therapeutic outcomes more so than feedback regarding client functioning more generally. The clinical implications of such a feedback tool, based on a questionnaire that is already routinely administered to our clients, are promising.

